# Supplementary material for: Baseline Characterization of the Gut Microbiota of Field and Colony Populations of Phlebotomus tobbi and Preliminary Assessment of the Anti-Leishmanial Activity of Cultivable Bacteria
Source: Pathogens. 2026 Jun 23;15(7):658. doi: 10.3390/pathogens15070658 (PMC13416292; doi:10.3390/pathogens15070658)
Supplement: Supplementary file 1 [file pathogens-15-00658-s001.zip › pathogens-4357168-File S1/Taxa2ASV_Decomposer/Genus/g__Pantoea/seqs.mltree.pdf]

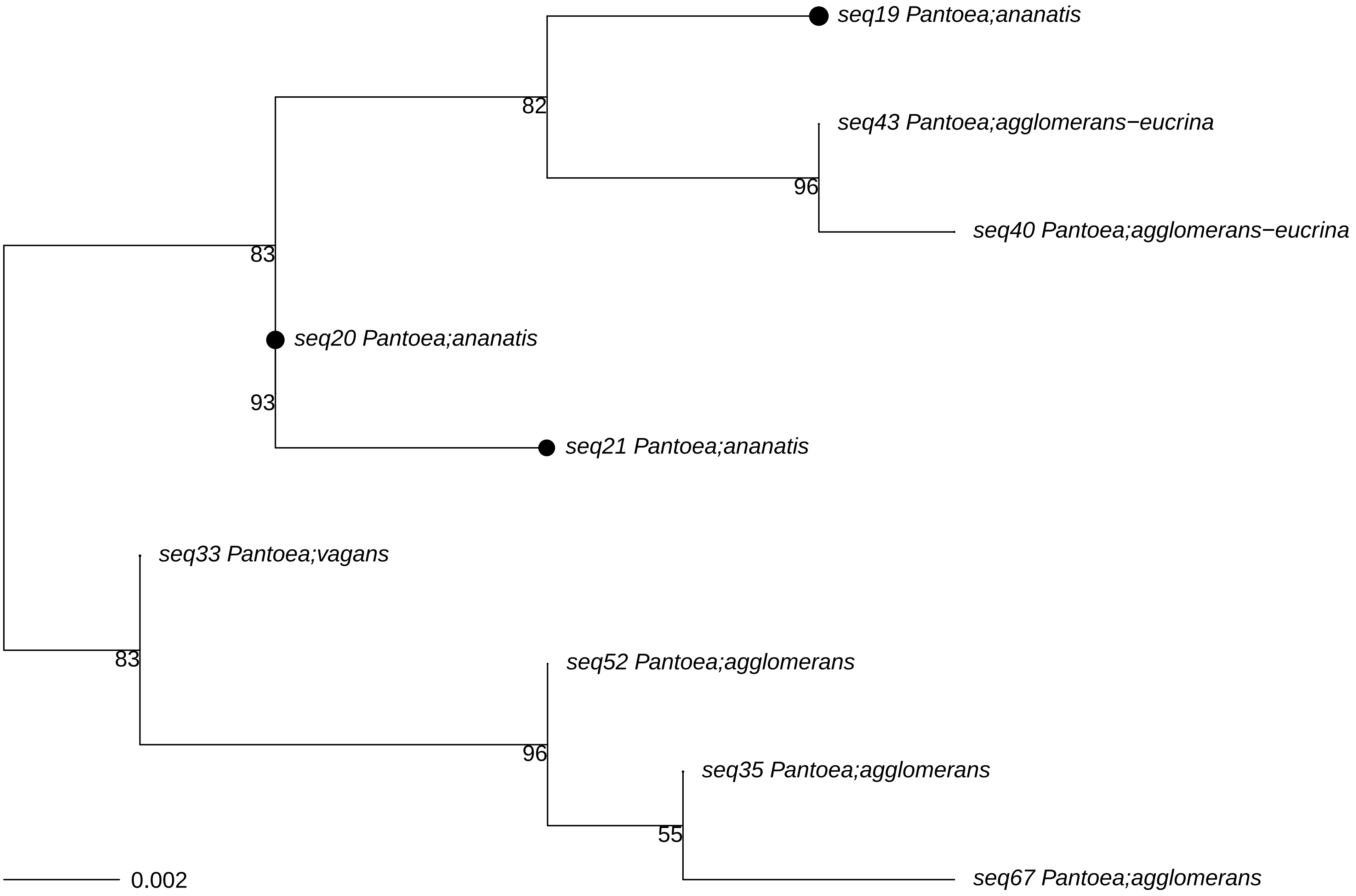
seq19 Pantoea;ananatis
82
seq43 Pantoea;agglomerans−eucrina
96
seq40 Pantoea;agglomerans−eucrina
83
seq20 Pantoea;ananatis
93
seq21 Pantoea;ananatis
seq33 Pantoea;vagans
83
seq52 Pantoea;agglomerans
96
seq35 Pantoea;agglomerans
55
seq67 Pantoea;agglomerans
0.002
